# Dietary Selenium and Human Health

**DOI:** 10.3390/nu9010022

**Published:** 2016-12-30

**Authors:** Lutz Schomburg

**Affiliations:** Institute for Experimental Endocrinology, Charité Medical University Berlin, Suedring 10, CVK, D-13353 Berlin, Germany; lutz.schomburg@charite.de; Tel.: +49-30-450-524289

**Keywords:** selenium, selenoprotein, micronutrient, essential trace element, supplement, nutrigenomics, selenosis, hidden hunger, biogeochemistry

## Abstract

Next year (2017), the micronutrient Selenium (Se) is celebrating its birthday—i.e., 200 years after first being identified by the Swedish chemist Jöns Jakob Berzelius. Despite its impressive age, research into the functions of this essential trace element is very alive and reaching out for new horizons. This special issue presents some recent fascinating, exciting, and promising developments in Se research in the form of eight original contributions and seven review articles. Collectively, aspects of Se supply, biochemical, physiological, and chemotherapeutic effects, and geobiological interactions are covered by leading scientists in the areas of nutritional, basic, and clinical research. It is obvious from the contributions that the bicentennial anniversary will celebrate a micronutrient still in its infancy with respect to being understood in terms of its biomedical importance.

Four of the contributions in the special issue discuss the difficulties in estimating, assessing and positively affecting the Selenium (Se) status. Gerald F. Combs reviews the problem of assessing and monitoring the Se status in human subjects; i.e., the quest for the best Se-dependent biomarkers in healthy or diseased and well- or poorly-supplied individuals, respectively. From the perspective of a European researcher, the impression prevails that both circulating selenoproteins and total serum or plasma Se concentrations provide adequate information on a given subject. However, the challenge from the perspective of a researcher residing in a Se-rich environment is different, as it is not only important to detect and define a Se deficit, but also to rate the Se status of sufficiently supplied subjects who are at risk of surplus Se intake [1]. The underlying reason for these two contrasting viewpoints is of course given in the relatively high Se intake in most areas of the US, where supplemental intakes raise concerns, whereas in many European, African, or Asian populations, the detection of a Se deficiency is the major issue. Besides discussing meaningful biomarkers, the review points to the fact that measuring Se or a circulating selenocompound or selenoprotein does not necessarily reflect the Se status in a given cell or tissue. It is only from model animals like rodents, chicken, or other species that we have data relating serum or plasma Se biomarkers with tissue and intracellular Se concentrations and selenoprotein expression levels. The data on humans are mainly restricted to total blood, serum, or plasma and hair, nails, blood, or buccal cells and urine, respectively. In general, we see that higher Se intakes do correlate to higher biomarker readings, until a certain level of saturation is reached for the circulating selenoproteins. At this point of supply, more research is needed to identify molecular readouts and to better characterize the metabolism of dietary selenocompounds and their effects on Se-responsive biomarkers distinct from the saturated selenoproteins.

The issue of deficiency constitutes the major topic in the systematic review by Rita Stoffaneller and Nancy L. Morse, who compile the available data on Se status in Europe and the Middle East [2]. Two major trends become obvious in this compilation. That is, the average Se intake is lower in East as compared to West European populations; and there is a wide variation of Se status, especially in the Middle East, likely reflecting different nutrition patterns and socioeconomic status of the populations. It is worrisome that the data from Turkey, Lebanon, or Saudi Arabia do provide evidence for a health-relevant Se deficit in a subset of individuals—especially in children. Yet, in view of the variations in the data reported from the same or neighboring areas, technical limitations need to be excluded before deducing far-reaching conclusions, and the use of standardized reference materials is encouraged in future analyses to allow a more detailed comparison. Nevertheless, additional studies—especially in the poorly-supplied populations—are warranted not to miss an easily avoidable disease risk factor, especially in the most vulnerable subjects (i.e., the children). However, it is not easy to successfully elevate the Se status of healthy subjects, as documented below.

Malene Outzen and colleagues attempted to efficiently raise the Se status of their Danish fellow citizens by encouraging an increased fish and mussel intake [3]. They conducted an open label intervention study enrolling participants with relatively low habitual dietary Se intake. The subjects in the supplementation group were invited to consume an extra 1 kg of fish and mussels per week for half a year, corresponding to an additional intake of approximately 50 µg Se/day (i.e., twice as high as the average basal Se intake in Denmark). The intervention resulted in the expected increase in both total blood Se and plasma selenoprotein P, albeit the efficiency was moderate, and the two biomarkers increased by an increment of only 14.9 µg/L and 7.0 µg/L, respectively. This is a relatively small effect and a partly disappointing finding, indicating that fish intake alone is insufficient to efficiently improve the Se status in middle-aged healthy subjects.

In contrast to these attempts on how to increase the average Se intake, Yujie Ning and colleagues question the necessity of continuing Se supplementation in the Kashin–Beck Disease (KBD) areas in China [4]. At present, selenite-enriched table salt (1:60,000) is provided and used regularly in the Se-deficient areas. The authors used a random sampling method with a 2:1:1 design; i.e., they matched two children in a Se-supplemented KBD area with one in a non-supplemented KBD area and one in a non-KBD area. They compared Se intake by a 24 h retrospective food frequency questionnaire, and determined Se in the hair of children 4–14 years of age. The Se status in the KBD areas was about 8% lower compared to hair samples from children in non-KBD areas. A similarly small difference of 5% was observed between male and female children. Interestingly, hair Se was higher in young (4.0–6.9 years) versus older (7–14 years) children. These data indicate that the Se deficit in the KBD areas is still present but relatively small, and that young children especially seem to be better supplied than in the past. As KBD starts in childhood and clinically manifests mainly in adulthood, the results are reassuring that effective prevention from severe Se deficiency is currently in place. The authors identify a number of dietary and non-dietary factors apart from Se-enriched salt that contribute to the Se supply, and conclude that the additional table salt supplementation may be dispensable. 

Another three contributions to this special issue describe the associations of Se intake and genetic background on chronic diseases, body fat, and therapy success in AIDS. Catherine Méplan summarizes the interrelation of Se and chronic diseases from a nutritional genomics perspective [5]. In her review, she reconciles the conflicting data from epidemiological and supplementation studies; i.e., a generally observed inverse association of low Se intake with increased risk for various chronic diseases, and a partial lack of supplementation effects observed in the respective intervention trials. Additional genetic factors may contribute decisively to some of this disparity and critically affect the relationship between Se status and health risks. Single nucleotide polymorphisms in the selenoprotein genes or in central genes implicated in Se metabolism or control of selenoprotein expression are one major modifier of Se effects. The studies that describe health effects of Se in relation to genotype imply that a nutritional genomics approach is contributing to a better understanding of the interaction of Se status and risk for multifactorial diseases. Moreover, the inclusion of genetic data may also help to uncover roles for particular selenoproteins in specific diseases or tissue function. Ultimately, this approach may lead to an improved and personalized dietary recommendation on Se intake, depending on the genetic makeup, Se status and other characteristics of a given subject (e.g., body weight and body fat).

This exact issue is highlighted by the astonishing results reported by Yongbo Wang and colleagues on the interaction of Se intake with markers of body fat [6]. The scientists analyzed a total of 3214 subjects enrolled in a cross-sectional study named the “Complex Diseases in the Newfoundland population: Environment and Genetics (CODING)”. They estimated the dietary Se intake by a specifically tailored food frequency questionnaire, measured circulating metabolic parameters and endocrine hormones, and determined body composition by the highly accurate method of dual-energy X-ray absorptiometry. As expected, daily Se intake was higher in males than in females. However, after correction for body weight, there was no difference between the sexes. Similarly, there was no interaction between Se intake and obesity in a direct analysis. Yet again, obesity was strongly associated with low Se intake when the intake was corrected for total body weight. The difference was surprisingly high, and Se intake per kg of body weight was on average 24% and 31% lower by obese subjects as compared to non-obese men and women, respectively. Notably, the body composition parameters BMI, waist circumference, as well as trunk, android, gynoid, and total body fat were lowest in the subjects with highest weight-based dietary Se intake, with a nearly linear dose-dependent inverse relationship. Dietary Se intake (μg/kg/day) alone accounted for 9%–27% of the variations in body fat, and an incremental increase in daily Se intake of 1 μg/kg/day corresponded to a 3%–6% decrease in body fat percentage. The associations were independent of age, calorie intake, physical activity, smoking, alcohol, medication, or menopausal status. The authors conclude that a high dietary Se intake is associated with a beneficial body composition, and a Se deficit may negatively affect food intake, metabolism, and the relative balance of fat and muscle tissue. As this notion applies to normal weight, overweight, and obese individuals, the better control of Se status may be a promising perspective for affecting the obesity endemic, given that these results can be verified in suitable intervention studies.

Rupak Shivakoti and colleagues present the latest data on the Se status in patients infected with HIV-1 from the large Prospective Evaluation of Antiretrovirals in Resource Limited Settings (PEARLS), a multi-country case–cohort study on the efficacy of antiretroviral therapy (ART) [7]. The authors defined Se deficiency as serum Se concentrations below 85 µg/L, which was present in about half of the patients enrolled, and then examined the risk factors for Se deficiency and the efficacy of antiretroviral therapy in relation to Se status. As expected, major risk factors for Se deficiency were previous diseases (tuberculosis, anemia, elevated C-reactive protein) and country of residence, as it is a major determinant of local soil Se concentrations and Se content of major food items. Unexpectedly, the authors observed that clinical failure to ART was highest in patients in the top tertile of serum Se. This finding contrasts to a number of prior studies reporting that mortality and morbidity are highest in the HIV patients with lowest Se status. The data question the protective and supportive role of high Se status during antiviral treatment and challenge the general idea that Se supplementation is always a health-supporting measure in diseased subjects during therapy.

From epidemiological and observational studies, it is only a small step to intervention trials with active Se supplementation, which are discussed in three more contributions. The article by Carina Benstoem and colleagues addresses an emerging issue in clinical research; i.e., the importance of Se in cardiovascular disease [8]. The observational and interventional studies published so far present a heterogeneous picture, with some associations of low Se status and higher cardiovascular disease risks and poorer disease courses, and other studies reporting null findings. The strongest evidence for a direct interaction is still given by the endemic cardiomyopathy found in the large Se-deficient region in China (Keshan Disease belt), where a Se deficit is a proven predisposing risk factor for the cardiomyopathy. It remains to be seen to what degree the interaction of cardiovascular health and treatment of heart diseases is related to the Se status and a sufficiently high supply. From the data at hand, it may be therapeutically of benefit to actively increase the Se status in the diseased subjects, both for reasons of prevention and as an adjuvant treatment during surgery; however, a sufficiently powered and well-controlled intervention trial studying this is unfortunately missing.

Ola Brodin and colleagues present the first results from the so-called SECAR trial; i.e., an intervention study with high dose selenite supplementation in cancer patients [9]. This phase I clinical study was not aimed at assessing the chemotherapeutic efficacy of selenite treatment, but rather to determine dose-limiting toxicities of selenite and defining the maximum tolerated dose as a prerequisite for such therapeutic applications. The rationale for this approach comes from preclinical studies reporting that high selenite exerts antitumor effects and increases efficacy of cytostatic drugs in model systems. In addition, tumors actively accumulate Se, and may thus constitute a more sensitive system for Se toxicity than neighboring untransformed cells. The theoretical background and the perspectives of this brave and unparalleled endeavor are scholarly summarized in a very instructive accompanying review by Sougat Misra and colleagues [10]. As one major and important result from the SECAR trial, the authors observed that toxic side-effects of brief high dose selenite supplementation were reversible and of short duration only. A second important result relates to the surprisingly high dosage of selenite that was well tolerated, far exceeding what would have been expected by the experts in the field, i.e., up to 10 mg per square meter body surface. In perspective, the study points to some promising treatment effects, including a direct reduction of tumor size and reversal of chemotherapy resistance in some of the treated patients. However, as this study was neither intended nor sufficiently powered to evaluate treatment effects, follow-up studies into this direction are eagerly awaited.

This special issue also contains some fascinating mechanistic studies on the effects of Se on selenoprotein expression, the immune system, and inheritance. Franziska Hiller and colleagues highlight the importance of the chemical form of supplemental Se for health-relevant effects of the micronutrient [11]. As an experimental model, the authors used mice with dextran sulfate-induced colitis and compared an organic versus an inorganic supplement. While both selenite and selenomethionine successfully increased selenoprotein expression rates and enzymatic activities of glutathione peroxidases and thioredoxin reductases, the intestinal inflammation remained unaffected by selenomethionine, but it was exacerbated in response to selenite treatment. These results are in contrast to expectation, as prior reports consistently observed anti-inflammatory effects of supplemental Se. Therefore, such pro-oxidative effects of short-term selenite treatment are noteworthy and highlight that selenocompounds not only affect the Se status by the micronutrient, but depending on the chemical backbone may also elicit tissue-specific Se-independent effects. Especially in view of the Se deficit that is often observed in inflammation or chronic gastrointestinal diseases, these results indicate that the form and route of supplementation is a relevant health factor in addition to the dosage of the given selenocompound.

The immune system apparently represents a most central and Se-sensitive target tissue. This notion is supported by numerous studies reporting Se-dependent infection rates, survival odds in sepsis, risk of autoimmune thyroid diseases, vaccination responses, genetic associations with cytokine concentrations, and reaction of model systems to experimental inflammation. Petra A. Tsuji and colleagues monitored the effects of a sufficient or limiting Se supply on the expression of cytokines, selenoproteins, and non-selenoproteins in the liver and lung of experimental mice [12]. Importantly, the authors combined a set of refined analytical techniques to obtain a most comprehensive picture, including ribosome profiling, deep sequencing, quantitative polymerase chain reaction (qPCR), and microarray expression analyses in combination with transcriptome shotgun sequencing RNA-Seq. The stress-related selenoprotein transcripts of Gpx1, Selh, Selk, and Sepw1 were identified as the most sensitive hepatic transcripts, whereas mRNA of the housekeeping selenoprotein genes Txnrd1, Gpx4, and Sephs2 were hardly affected by the Se status. Ribosome footprint analyses identified the areas around the Sec codons as being particularly enriched (i.e., occupied by ribosomes), verifying the deceleration of ongoing translation at Sec insertion sites. In combination with the RNA-Seq data, a unique view on the ribosome traffic jam around this most fascinating translational step at the UGA codon is presented, shedding a bright molecular light on the recoding miracle, and the constant challenge a ribosome is facing by each selenoprotein transcript.

A thought-provoking study presented by Matthew P.G. Barnett and colleagues indicates that a low Se intake may also affect the next generation [13]. The team compared groups of mice receiving a diet high in fat and supplemented or marginally deficient in folate and Se. The diets were given to female mice during pregnancy and/or weaning, and importantly to the offspring after weaning. Then, gene expression patterns in liver and colon of the male offspring were analyzed and compared. The results indicate that Se and folate supply to the mothers had a persisting impact on the expression of key metabolic genes in the livers of the offspring, and that the effect via the mothers was of even greater magnitude compared to the same diets given directly to the offspring after weaning. These findings emphasize the importance of the micronutrient and vitamin supply during pregnancy (and importantly during early pregnancy), especially in view of the notion that a diet high in fat, calories, and macronutrients does not necessarily cover the requirement for trace elements and vitamins (please see the CODING study above). This lack of essential micronutrients in a high caloric diet is known as “hidden hunger” and may constitute an important parameter in today’s global obesity and diabetes endemic.

The last two contributions to be highlighted in this editorial address special aspects of nutritional sciences—i.e., a global and a subcellular perspective. Alan M. Diamond discusses the importance of protein–protein interactions and subcellular localization in controlling selenoprotein activities [14]. It is not only in histology that location is of utmost relevance, but also in enzymology and signaling, where many proteins only exert their biochemical functions after having reached their subcellular destiny. The author chooses two selenoproteins as highly instructive examples (i.e., GPX1 and GPX4), along with an intracellular Se-binding protein, SeBP1. GPX4 is known as a moonlighting selenoenzyme, being converted during gamete maturation into an enzymatically inactive polymer providing strength and structure to sperm. Moreover, differently spliced GPX4 transcripts encode nuclear, cytosolic, or mitochondrial protein variants. Similarly, GPX1 has been identified in both mitochondria and cytosol. Interestingly, GPX1 is capable of forming a direct complex with the Se-binding protein SeBP1, thereby the expression and activity levels of the partner protein are negatively affected. This interaction of two Se-related proteins regulates their activities and may also control their subcellular localizations, which again may be of clinical relevance as the expression has been associated with tumor biology, cancer stage, and disease prognosis, thereby providing another potential link to the nutritional genomics and SECAR study data mentioned above.

Finally, Lenny H.E. Winkel and colleagues expand our appreciation of our favorite micronutrient by providing a global perspective on Se cycling between the abiotic and living world; i.e., from soils to plants and animals and back again via microorganisms, air, rain, and other routes into the earth, ponds, rivers, and oceans [15]. This review takes the reader on a highly inspiring intellectual and scientific voyage across the different disciplines of science, from geology via plant biology, genetic engineering, physiology, and medicine to the atmosphere, lithosphere, and hydrosphere, for which the term "biogeochemistry" has been coined. Humans do cover a large part of their essential Se supply by consuming plants for which the trace element is not essential. Still, plants may profit from Se uptake in several ways; becoming protected from herbivory attack and other pathogens, benefiting via elemental allelopathy, and escaping from competition with other vegetation. Consequently, the Se status in humans is tightly regulated and relatively uniform across the populations, whereas plants differ considerably and may contain a range from nearly zero to enormously high Se concentrations, depending on species, plant part (roots, leaves, or fruit), and soil quality. Some Se hyperaccumulators native to seleniferous soils accumulate up to 15,000 mg Se/kg dry weight; which is not only of interest for dietary exploitation, but also for remediation of Se poisoned soils. As Se deficiency is a major issue in large areas of Africa, Asia, and Europe, the issues of Se cycling across the soil–plant–atmosphere interfaces and the attempts of biofortification by actively enriching the agricultural products with Se are scholarly discussed. While the main anthropogenic source of Se is coal combustion, marine organisms contribute the largest fraction to the atmosphere by evaporating methylated Se metabolites. Understanding the atmospheric and geogenic sources of Se will help to better control average Se intake of whole populations. This is nicely exemplified by the Se-poor belt in China, where the Se status is decisively affected by the monsoonal precipitations. A more refined knowledge of these processes will allow for an improved understanding of Se distributions in the future, which helps control the Se-related health hazards to humans, animals, and plants.

Collectively, this special issue draws a most colorful, multifaceted, and promising picture of the current research on Se and selenoproteins in the nutritional sciences (Figure 1). It highlights that many different disciplines contribute to our current appreciation of this essential trace element as a most fascinating and rewarding topic of interest and dedication.

## Figures and Tables

**Figure 1:**
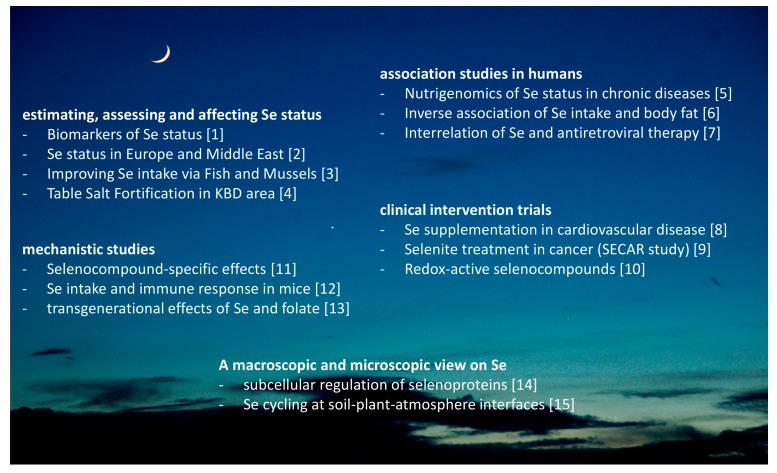
Overview on the Special Issue entitled “Dietary Selenium and Human Health”. The topics covered in the special issue range from the microscopic to a global perspective, from association to intervention studies in both humans and model systems, with the aim of providing an up-to-date insight into the various research fields that nowadays contribute to our understanding of this essential trace element 200 years after its identification. The numbers in brackets refer to the references. The photograph showing the moon and implying that our work is conducted under the grace of Selene was kindly provided by Wyck Hoffler, MD, Titusville, FL, USA.
